# Time of Day and Chronotype-Dependent Synchrony Effects Exercise-Induced Reduction in Migraine Load: A Pilot Cross-Over Randomized Trial

**DOI:** 10.3390/ijerph20032083

**Published:** 2023-01-23

**Authors:** Elias M. Malek, James W. Navalta, Graham R. McGinnis

**Affiliations:** Department of Kinesiology and Nutrition Sciences, University of Nevada Las Vegas, Las Vegas, NV 89154, USA

**Keywords:** exercise, circadian, chronotype, migraine, headache, chrono-exercise, time-of-day

## Abstract

Migraines are the most common cause of chronic pain. Effective, non-pharmacological strategies to reduce migraine load, like exercise, are needed, but it is unclear how exercise timing and chronotype modulate the effects. We sought to determine the effects of time-of-day of exercise, and synchrony with one’s chronotype, on migraine load. We performed a pilot cross-over randomized trial where participants with chronic migraine completed two one-month exercise interventions, consisting of either morning exercise (before 09:00 a.m.) or evening exercise (after 7:00 p.m.) in a randomized repeated measures cross-over design (Clinical Trial #NCT04553445). Synchrony was determined by exercise time and chronotype (i.e., a morning type participant exercising in the morning is ‘in-sync,’ while an evening type participant exercising in the morning is ‘out-of-sync’). Migraine burden, and anthropometric assessment occurred before and after each month of exercise. Data was analyzed using repeated measures ANOVA with significance accepted at *p* < 0.05. When comparing morning and evening exercise, there was no significant improvements in any migraine-related parameters. However, when comparing in-sync and out-of-sync exercise, we found that migraine burden was only improved following in-sync exercise, while no benefits were seen in out-of-sync exercise. Our data suggests that exercise timing has limited impact, but synchrony with chronotype may be essential to decrease migraine load in chronic migraineurs.

## 1. Introduction

Chronic pain affects millions of US adults each year, with chronic migraines (CMs) being one of the most common forms [1,2,3]. Migraine imparts a large physical and financial burden, costing the US $560–635 million annually [2], as well as considerable loss in productivity at work [4]. Migraine is a cyclic disorder accompanied by headache-like symptoms, as well as potential photophobia, phonophobia, and transient focal neurological symptoms [5]. The International Headache Society (IHS) classifies CM as headaches (migraine-like or tension-type-like) ≥15 days per month for ≥3 months, where ≥8 episodes have migraine-like features [5]. Current treatment of CM involves multiple modalities, including pharmacological treatment (e.g., calcitonin gene-related peptide (CGRP) inhibitors, analgesics), as well as various non-pharmacological interventions (e.g., cognitive behavioral therapy, meditation or stress management, and exercise). While modern pharmacological treatments have shown increasing efficacy, some may induce various side effects, leading to increasing interest in alternative treatments with lower adverse effects [6].

Exercise is likely the most accessible non-pharmacological intervention capable of reducing migraine load in people with CM. Exercise interventions, including aerobic or resistance training, have been shown to decrease migraine frequency, pain, duration, and the number of migraine days [3,7,8,9], which could decrease use and reliance on prescription drugs. In fact, Varkey et. al. found that 71% of people with CM decreased the use of medicine to treat their migraines after six months of exercise [10]. Beyond reducing migraine burden, exercise also alleviates common comorbidities of migraine, such as depression, anxiety, and cardiovascular disease [11,12]. One perplexing issue is that aerobic exercise does not decrease migraine symptoms in all people with chronic migraine. In one study, 10 weeks of aerobic exercise performed by sedentary people with chronic migraine decreased the mean migraine frequency, pain intensity, and duration [13]. However, approximately 60% of participants responded, and the other 40% of participants were classified as non-responders, because they did not experience a ≥ 50% decrease in a migraine-related outcome [13]. The cause of non-response is unclear. While exercise has shown efficacy, scarce research has focused on important mediators of exercise prescription, including circadian timing (i.e., what time of day to exercise) and chronotype (i.e., preference for mornings/evenings).

Circadian rhythms are recurring ~24-h cycles of physiological processes, allowing temporal synchrony with our environment. These rhythms are driven by endogenous (the transcriptional feedback loop, known as the circadian clock) and exogenous (environmental cues, like light) factors, and synchronized by a specialized brain region called the suprachiasmatic nucleus (SCN) [14]. Recent research indicates that migraines originate in the hypothalamus, the same brain region that houses the SCN, suggesting a link between migraines and circadian rhythms [15,16]. In support, migraines have been shown to display circadian rhythmicity, with a peak in the morning and mid-day [17], which differs based on an individual’s circadian preference, or chronotype [18].

The time of day of exercise has previously been shown to modulate the perceptual, physiological, and biochemical responses to exercise [19,20,21,22,23], which may impact the adaptations or benefits of exercise training. A recent systematic review determined that consistent morning exercise facilitated greater exercise adherence and weight loss when compared to evening exercise in obese patients [24]. However, the response to acute exercise performed at different times of day are dependent on chronotype (i.e., evening types perform poorly in the morning compared to morning types) [21,25,26,27]. Perceptual and physiological responses [28,29] in response to acute exercise have been shown to be modulated by chronotype. However, no studies to date have investigated time-of-day dependent exercise prescription in the context of CM. Understanding this relationship introduces a novel advance to exercise as a treatment to reduce migraine burden. As such, the purpose of this study was to determine if morning exercise or evening exercise more potently reduced migraine load. Additionally, we assessed if the response was modulated by chronotype, to determine if performing exercise ‘in synchrony’ (IS) with one’s chronotype was more effective.

## 2. Materials and Methods

### 2.1. Participants and Trial Design

**Figure 1:**
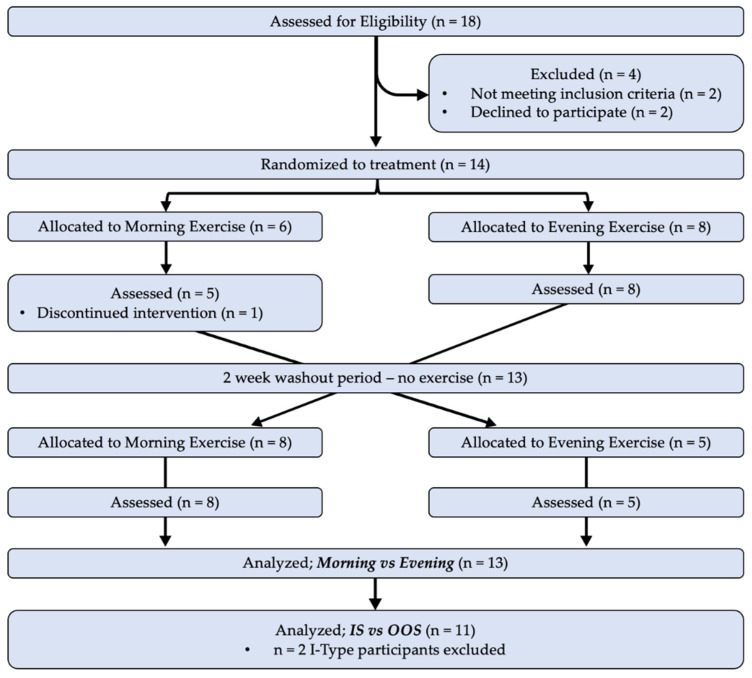
CONSORT diagram outlining the enrollment, allocation, and analysis of participants included in this study. TOD—Time-of-day.

Healthy sedentary participants aged 18–55 years, who self-identified as CM (8+ migraines/month), but were otherwise healthy, were recruited to participate in this pilot cross-over randomized trial, which was guided by the Consolidated Standards of Reporting Trials (CONSORT) extension for crossover studies [30] (see Supplementary Table S1 for CONSORT Checklist). Inclusion criteria for participants in this trial were that they were not recreationally active prior to beginning their participation in the study, were non-smokers, not pregnant, and had no history of cardiac, renal, pulmonary, musculoskeletal, or metabolic disease. Participants were randomly allocated in an alternating counterbalanced fashion to complete four weeks of morning exercise (ME) or evening exercise (EE) during the initial phase of the study period, with assessments at baseline and at the conclusion of each exercise period. Participants then completed a two-week washout period where they did not participate in any programmed exercise, at which point they were crossed over to complete the alternate period of exercise (e.g., phase 1: ME, phase 2: EE; see ‘Exercise’ section below, and CONSORT diagram in Figure 1). All data were collected in the research laboratory of the investigators, with the exception of physical activity tracking of exercise (performed remotely and logged digitally, see Section 2.2). Due to the intentional nature of performing exercise in the morning or evening, it was not possible to blind participants to their group allocation. Members of the research team were not blinded to the time of day that participants were prescribed to exercise, and participant allocation was not concealed from investigators prior to, or during, randomization. Although this presented a limitation to our study design, the risk of bias was reduced by performing a cross-over design, as participants completed both exercise prescriptions. Recruitment and performance of this trial took place from December 2020–April 2022.

### 2.2. Exercise Interventions

Participants completed one month of morning exercise (ME; before 9:00 a.m.) or evening exercise (EE; after 7:00 p.m.) in a randomized, counterbalanced, cross-over design with a two-week wash-out period. The exercise prescription was of moderate exercise intensity (60–70% age-estimated maximal heart rate; 220-age), and a duration and frequency that beginning exercisers could tolerate and perform [31]. Exercise frequency and duration were prescribed at ≥3 days/week and ≥30 min/session, respectively, but participants could exercise more if desired. Participants were provided with a list of self-selected exercise activities to choose from (i.e., walking, running, cycling, jumping rope, calisthenics, etc.), but modality was not prescribed. Time-of-day of exercise, exercise intensity (average heart rate), duration, and adherence ([# sessions completed]/12) were determined from the exercise log (recorded in Polar App). The exercise prescription and recommendation were identical for both times of day.

### 2.3. Assessments and Laboratory Visits

Participants completed four laboratory assessments, namely, at the beginning (pre-) and end (post-) of each exercise condition. Assessments included the completion of perceptual questionnaires and participant anthropometric characteristics. Height was obtained using a stadiometer, and body mass and composition (lean/fat mass %) were determined using a SECA medical Body Composition Analyzer 514 (SECA Deutschland, Hamburg, Germany). Participants were provided with a heart rate monitor (Polar H-9 heart rate sensor, Polar USA, Lake Success, NY, USA), and trained on fit, usage, and connection with the Polar Beat App via Bluetooth (installed on each participant’s phone). Completion of all exercise sessions was recorded using the heart rate monitor and app allowing the research team to assess adherence to the exercise prescription (including intensity, duration, and frequency).

### 2.4. Questionnaires

At baseline, participants completed the Current Exercise Training Questionnaire to confirm sedentary status, and the Morning/Evening Questionnaire (MEQ) to determine their chronotype (categorized as Morning-; M-Type, Intermediate-; I-Type, or Evening Type; E-Type). Our primary outcome was migraine load, which was evaluated using the Headache Impact Test (HIT-6) and Migraine Disability Assessment Test (MIDAS), both of which are valid and reliable tools for measuring the impact of chronic migraines [32,33]. MIDAS scores were reported as a value between 0 and 21 with migraine days and migraine pain intensity evaluated separately. For both questionnaires, lower scores indicated lower migraine burden. Participants completed HIT-6 and MIDAS a total of four times, at the beginning and end of both exercise interventions.

### 2.5. Responder Status

We utilized minimally important change (MIC) thresholds for migraine burden to categorize participants as responders and non-responders. The within person MIC was reported to be 2.5 points for the HIT-6 questionnaire in [34], and 4.5 points for the MIDAS questionnaire in [35]. As such, if a participant’s HIT-6 or MIDAS score decreased as a result of exercise by 2.5 or 4.5 points, respectively, they were designated ‘responders.’

### 2.6. Statistical analysis

Utilizing data from a previously published study utilizing exercise intervention resulting in a significant reduction in migraine days (Kroll et al. [3]), an effect size of 0.71 was calculated. A power analysis based on this effect size (α = 0.05, β = 0.80) revealed a targeted sample size of 14 participants. Eighteen participants were initially recruited, 14 participants were allocated to treatment during the first month, and 13 participants completed the entire study.

The effects of the time of day of exercise were assessed by comparing Pre- and Post-values from the periods of Morning Exercise (ME) vs. Evening Exercise (EE). We also performed a secondary analysis to test the contribution of participant chronotype on the effects of morning or evening exercise. For the secondary analysis, the time of day of exercise and participant chronotype were grouped into two separate subgroups; In-Sync (IS), were participants who exercised at the time of day most aligned with their chronotype, and Out-of-Sync (OOS), were participants who exercised at a time of day *misaligned* with their chronotype. For example, M-Type participants performing morning exercise, and E-Type participants performing evening exercise were considered ‘IS.’ M-Type participants performing evening exercise, and E-Type participants performing morning exercise were considered ‘OOS’ (I-Type were excluded from this analysis).

When comparing the average pre- to post-change in outcomes between ME and EE, or IS and OOS, a paired samples *t*-test was used. To assess the effects of the time-of-day of exercise (ME vs. EE) and synchrony with chronotype (IS vs. OOS) on the ability to reduce migraine load, a 2 × 2 within-subjects repeated-measures ANOVA was used. Differences in migraine load across the months of ME and EE were assessed with the ANOVA model with fixed factors in terms of time-of-day (ME vs. EE) and exercise (pre- vs. post-), as well as an interaction. The same analysis was completed for IS and OOS exercise with synchrony and exercise set as fixed factors. Values were reported as mean ± SEM unless otherwise specified. Effect size (ES) from repeated measures ANOVA was reported as partial Eta squared (η_p_^2^), with 0.01 representing a small effect size, 0.06 representing a medium effect size, and over 0.14 representing a large effect size [3]. If the interaction was significant, differences were determined using paired samples t-tests. Effect sizes from paired samples t-tests were reported as Cohen’s *d* with 0.2 indicating a small effect size, 0.50 representing a medium effect size, and over 0.80 indicating a large effect size [3]. The distribution of responders and non-responders was evaluated using chi squared analysis (χ^2^). The expected distribution, with respect to in-sync exercise, was compared relative to the percentage observed for out-of-sync exercise. In all cases, significance was accepted at the *p* ≤ 0.05 level. Analyses were conducted using SPSS version 28 (IBM, New York, NY, USA). In all cases, *p* < 0.05 was considered statistically significant.

## 3. Results

The study population is outlined in a CONSORT diagram (Figure 1), which details the participants included in the Time-of-Day analysis (ME vs. EE), and the Synchrony analysis (IS vs. OOS). Of the 18 participants screened for eligibility, 14 participants were randomized to treatment, and 13 completed the entire protocol (*n* = 11 females, *n* = 1 nonbinary, *n* = 1 male). There were no changes to the interventions or primary outcomes during the study. Study recruitment was stopped when we achieved our target sample size, though one participant did not complete the intervention. Demographic characteristics for the participants are included in Table 1. In the analysis of ME vs. EE, *n* = 13 participants completed all aspects of the study and were included. For the comparison of IS vs. OOS exercise, *n* = 11 participants were included in the analysis. Two participants were excluded from IS vs. OOS analysis due to their chronotype being ‘intermediate’ (I-Type). With an intermediate chronotype, ‘synchrony’ with ME or EE could not be determined.

We did not have any adverse events, such as injuries, falls, etc., during the study period. Only one participant was unable to complete the exercise intervention, which was due to lack of interest in continuing the intervention.

### 3.1. Exercise Performance and Adherence

#### 3.1.1. Time-of-Day

**Figure 2:**
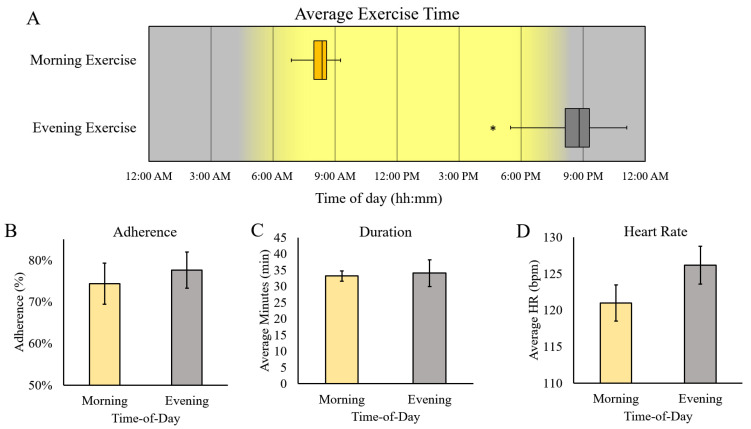
Implementation of TOD-Dependent exercise intervention. Representation of average exercise timing (**A**) and adherence (**B**) for ME and EE. Exercise session duration (**C**) and heart rate (**D**) were similar in both conditions. * Different from ME, *p* < 0.05.

The median exercise time for ME was 08:22 AM, and EE was 08:08 PM, indicating successful discrepancy in exercise timing (Figure 2A). There was no difference in adherence between ME and EE (74% ± 5% vs. 78% ± 4%, respectively; *p* = 0.35). The average exercise duration (ME = 33.2 ± 1.6 min, EE = 34.1 ± 4.2), and heart rate (ME = 121 ± 2 bpm vs. EE = 126 ± 3 bpm) were also not different, based on the TOD of exercise.

#### 3.1.2. Synchrony

**Figure 3:**
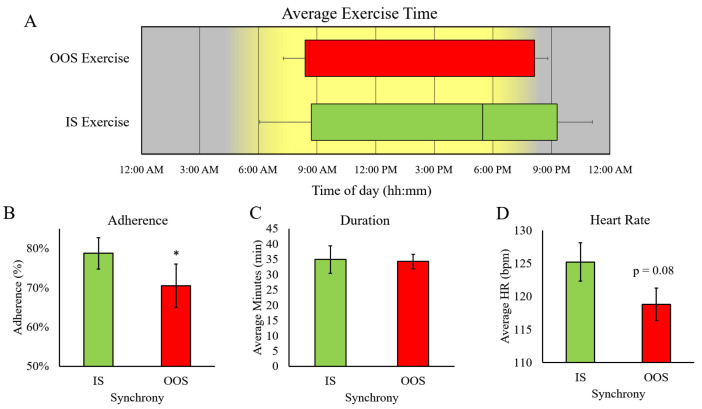
Implementation of IS vs. OOS exercise intervention. Representation of average exercise timing (**A**) and adherence (**B**) for ME and EE. Exercise session duration (**C**) and heart rate (**D**) were similar in both conditions. * Different from IS, *p* < 0.05.

Exercise timing during IS and OOS exercise was distributed evenly throughout the day, with the range of exercise times for IS exercise being between 06:52 AM and 11:05 PM and OOS exercise being 7:19 AM and 9:16 PM (*p* = 0.92) (Figure 3). Interestingly, we observed significantly higher adherence during IS exercise (IS = 79 ± 4% vs. OOS = 70 ± 5.5%, *p* = 0.03). The average exercise duration (IS = 34.8 ± 5 min vs. OOS = 34.2 ± 2.6 min) and HR (IS = 125 ± 3 bpm vs. OOS = 119 ± 2 bpm) were not significantly different between IS and OOS exercise.

### 3.2. Migraine Burden

#### 3.2.1. Time-of-Day

**Figure 4:**
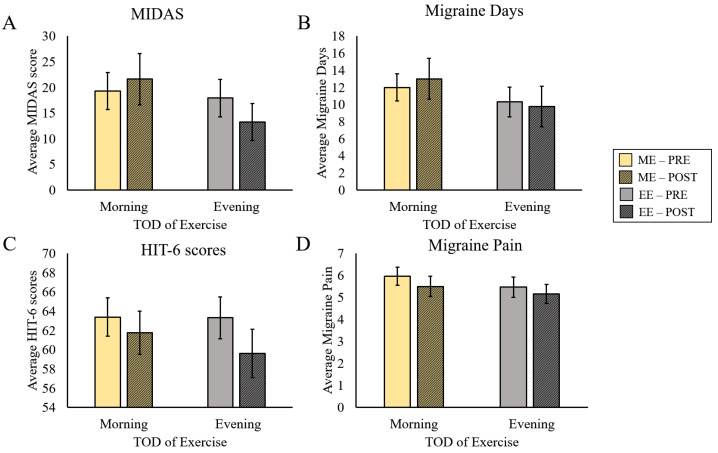
Effects of Morning or Evening Exercise on Migraine Load. Changes in migraine load between ME and EE for (**A**) average MIDAS scores, (**B**) average migraine days, (**C**) average HIT-6 scores, and (**D**) average migraine pain.

There was no significant effect of exercise on MIDAS scores (F(1,12) = 0.23, *p* = 0.64, η_p_^2^ = 0.02) and no interaction effect between exercise and TOD (F(1,12) = 2.89, *p* = 0.12, η_p_^2^ = 0.194) (Figure 4A). Similarly, there was no effect of exercise (F(1,12) = 0.03, *p* = 0.87, η_p_^2^ = 0.002), and no interaction between exercise and TOD in Migraine Days (F(1,12) = 1.39, *p* = 0.26, η_p_^2^ = 0.104) (Figure 4B). HIT-6 scores tended to decrease with exercise (Main Effect for Exercise; (F(1,12) = 3.42, *p* = 0.09, η_p_^2^ = 0.28, small ES)), although the effect did not reach statistical significance. There was no interaction between Exercise and TOD in HIT-6 scores (F(1,12) = 0.56, *p* = 0.47, η_p_^2^ = 0.05) (Figure 4C). Likewise, Migraine Pain did not exhibit any significant effects, though the Main Effect of Exercise approached significance (F(1,12) = 0.059, *p* = 0.06, η_p_^2^ = 0.005). (Figure 4D).

#### 3.2.2. Synchrony

**Figure 5:**
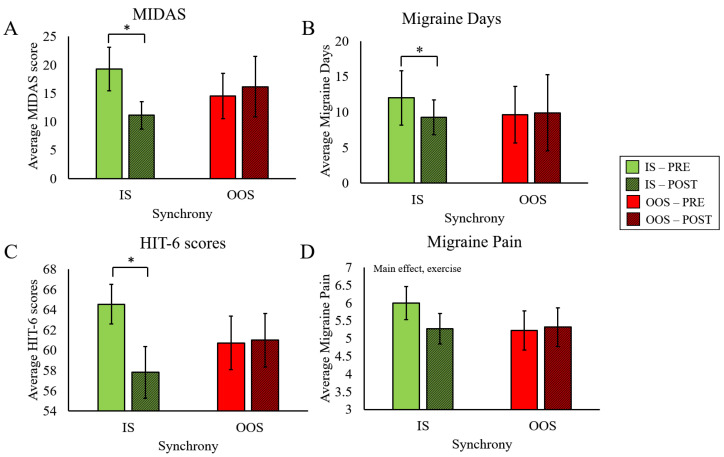
Effects of In-Sync or Out-of-Sync Exercise on Migraine Load. Changes in migraine load between IS and OOS exercise for (**A**) average MIDAS scores, (**B**) average migraine days, (**C**) average HIT-6 scores, and (**D**) average migraine pain. * different from PRE, *p* < 0.05.

We found a significant interaction between Exercise and Synchrony for MIDAS scores (F(1,10) = 14.6, *p* = 0.003, η_p_^2^ = 0.59), revealing significant improvement only after IS exercise (t(10) = 3.32, *p* = 0.004, Cohen’s d = 1.001) (Figure 5A). There was also a significant interaction effect for Migraine Days (F(1,10) = 5.76, *p* = 0.037, η_p_^2^ = 0.37), such that number of migraine days only decreased after IS exercise (t(10) = 2.83, *p* = 0.009, Cohen’s d = 0.85) (Figure 5B). Similarly, a significant interaction effect was present in HIT-6 scores (F(1,10) = 8.22, *p* = 0.02, η_p_^2^ = 0.45, small ES), where HIT-6 scores only improved after IS exercise (t(10) = 2.82, *p* = 0.009, Cohen’s d = 0.85) (Figure 5C). There was a near significant trend for the reduction of Migraine Pain in response to exercise (Main Effect Exercise, F(1,10) = 4.93, *p* = 0.051, η_p_^2^ = 0.33,, small ES). However, there was no interaction effect for Migraine Pain (F(1,10) = 1.47, *p* = 0.25, η_p_^2^ = 0.13) (Figure 5D).

**Figure 6:**
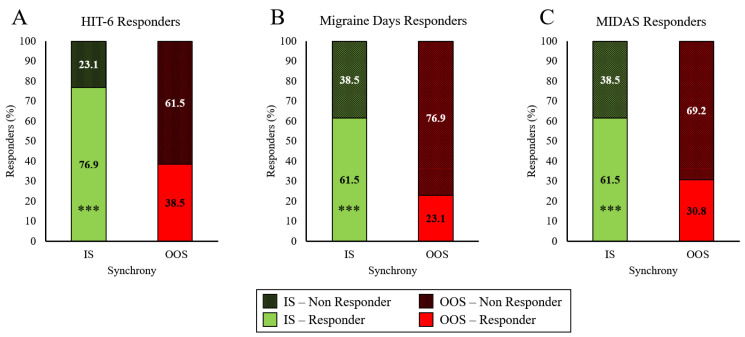
Percent of people with chronic migraine (*n* = 13) classified as responders (green) and non-responders (red) after completing 1-mo of exercise IS and OOS with the circadian preference, according to (**A**) HIT-6 score, (**B**) MIDAS score, (**C**) migraine days. *** *p* < 0.001.

To extend these results into clinical utility, we also present the exercise-induced reduction in migraine load in the context of surpassing the minimally important change (MIC) thresholds (“Responder”), and those that did not (“Non-Responder”). IS exercise resulted in a greater percentage of participants classified as responders on the HIT-6 questionnaire, compared with OOS (Figure 6A), (χ^2^ (1, *n* = 13) = 200, *p* < 0.001). Similar to the HIT-6, IS exercise resulted in more participants classified as responders on the MIDAS, when compared with OOS (Figure 6B), χ^2^ (1, *n* = 13) = 200, *p* < 0.001). When the number of migraine days per month was considered, IS exercise resulted in a greater percentage of participants classified as responders than OOS exercise (Figure 6C), (χ^2^ (1, *n* = 13) = 200, *p* < 0.001).

## 4. Discussion

This is the first study to investigate time-of-day and chronotype-dependent regulation of exercise-induced benefits in people with CM, and we identified a potentially important role for exercising in synchrony with chronotype. While a growing number of studies investigate exercise timing, very few account for the chronotype of participants. In a recent systematic review conducted by Vitale et al., they found only ten published articles that examined the effect of chronotype on exercise performed at different times of day. None of the papers reported in this review had participants exercise over a longer period than one day [26]. More recently, Thomas et al. conducted a study over 5=five days of exercise and found that exercise-induced circadian phase shifts were stronger when exercising out of sync with chronotype [20]. In the current study, we found that migraine outcomes were improved after one month of IS exercise, while no improvements occurred after OOS exercise. As such, it is possible that migraine improvement was related to strengthening circadian rhythms; however this assertion requires further study.

Although participants were instructed to complete at least three 30-min exercise sessions each week, we only had moderate adherence (~75%, not different between ME and EE, but slightly higher for IS compared to OOS). This was, indeed, interesting from a practical standpoint, and emphasized a novel way in which exercise adherence may be improved. This constituted a relatively mild exercise intervention that fell near, or below, generally recommended exercise prescriptions to see health benefits (ACSM). However, this exercise dose was sufficient to induce clinically meaningful reductions in migraine load. We found no effects of any exercise condition (ME vs. EE, or IS vs. OOS) on exercise intensity (based on average HR) or duration, though HR tended to be higher during EE and IS exercise (not significant). As such, exercise-induced improvements to migraine load observed in the current study were independent of overt differences in the exercise prescription.

### 4.1. Time-of-Day

In the current study, we did not observe a TOD-dependent effect of exercise on improving MIDAS scores, HIT-6 scores, and migraine pain. Exercising regularly has been shown to have positive therapeutic outcomes without causing side effects [7] and specific aerobic exercise protocols have been designed and validated to increase aerobic capacity without exacerbating migraine load, and, in many cases, migraine load was improved [10]. These findings supported our data where HIT-6 scores tended to improve after exercise (statistical trend). This improvement in HIT-6 scores was not influenced by the TOD at which exercise was performed, suggesting that the improvement could be attributed to exercise alone. The heterogeneity of response to exercise training suggests that participants’ chronotype might have a stronger influence on the physiological response to exercise than the time of day at which exercise was performed.

### 4.2. Synchrony

After analyzing the effect of TOD on exercise, we subsequently classified both exercise times as in-sync or out-of-sync with chronotype to adjust for preference. Interestingly, adherence to the exercise prescription during IS exercise was significantly higher than OOS exercise. To our knowledge, this is the first study to evaluate adherence to an exercise program for people with CM in this way and represents a clinically impactful tool to improve exercise prescription. These findings suggested that people with CM are more likely to comply with an exercise prescription if the exercise is prescribed at a time befitting their temporal preference. Previous studies have shown that people who adhere to exercise are able to manage their migraines in a more capable and confident manner [36,37]. While exercise session duration was similar between both IS and OOS months, heart rate tended to be higher during IS exercise (*p* = 0.07). It is possible that this trend represents a higher physiological response to exercise during a preferable time. As such, exercising at the time of day most aligned with a person’s chronotype may be more efficacious. Previous studies have shown that the perceptual response, via RPE, is also more favorable to exercise in sync with chronotype [26], providing further evidence that timing and chronotype are important considerations for exercise prescription.

When evaluating migraine burden, only IS exercise was capable of eliciting improvements, while OOS exercise had no effects. MIDAS scores improved after the month of IS exercise, but scores slightly increased after OOS exercise, with the same pattern being observed in HIT-6. Additionally, the number of migraine days experienced during the month of IS exercise decreased compared to baseline, while a minor increase was seen during OOS exercise. To our knowledge there are no previous studies investigating the effects of IS or OOS exercise *training* in healthy individuals or people with CM. Acute bouts of ME and EE have previously been shown to effect M-Type and E-Type participants’ differently [25,26,27]. For example, post-exercise vagal reactivation has also been shown to be modulated by synchrony with one’s chronotype [25]. These findings aligned with our hypothesis that exercising IS with one’s chronotype reduces migraine burden when compared to exercising OOS. The results from the present work indicate that when using exercise as an intervention to reduce migraine load in people with CM, it may be important to determine the patient’s chronotype and prescribe exercise in-synch with their chronotype to elicit any improvements.

### 4.3. Limitations

While our study followed a rather rigorous cross-over randomized design, several limitations existed. First, our participants with CM were self-diagnosed (via pre-established cut off criteria for the number of migraine days per month). We may have observed stronger changes if we had followed stricter inclusion criteria, including a CM diagnosis from a neurologist. Secondly, with our small sample size, we completed an intent-to-treat analysis rather than excluding participant data based on an a priori adherence rate. Our findings still indicated improvements in migraine load, even with moderate adherence. Third, we did not attempt to blind the participants or investigative team, and did not use sophisticated methods for allocation concealment, randomization, or blocking. Rather, participants were alternately assigned to either time of day for their primary exercise period. However, we did not see any effect of the time of day of exercise until we included the participants’ chronotypes. Two other mitigating factors further minimized potential bias stemming from an unblinded research team. First, in the secondary analysis, neither participants, nor investigators, were aware of participant MEQ scores (chronotype) or their synchrony with either time of day of exercise prescription. Secondly, our primary outcomes were perceptual questionnaires completed by the participants independently. As such, there was little to no input from the research team. Our last limitation is that we did not control for other lifestyle factors, such as diet and sleep, which have known impacts on migraine. Future clinical trials should be designed with these limitations in mind.

## 5. Conclusions

While exercise is known to reduce migraine load, and there is a well appreciated influence of circadian rhythms on the response to exercise, there are no studies investigating the efficacy of exercise as an intervention based on the TOD the exercise is performed in people with CM. This is the first study to find that exercise at a time of day in synchrony with chronotype improved the efficacy of an exercise prescription. In particular, migraine burden was only shown to improve through the month of IS exercise, while the same exercise performed out of sync had no benefit. Based on these findings, future, larger scale clinical trials are needed to examine the effects of chronotypical synchrony as a critical mediator of the efficacy and adherence to chronic exercise prescriptions in people with chronic migraine, as well as with other chronic conditions.

## Figures and Tables

**Table 1:** Demographic and anthropometric data for participants completing exercise intervention.

	Age (yrs)	Height (m)	Body Mass (kg)	M/F/NB	M-/I-/E-type
Mean ± SD	29.5 ± 11.5	1.67 ± 0.05	84.8 ± 28.6	1/11/1	5/2/6

NB—Non-binary, M-Type—Morning Type, I-Type—Intermediate Type, E-Type—Evening Type.

## Data Availability

Original research data is available directly from the authors upon reasonable request.

## Supplementary Materials

The following supporting information can be downloaded at: https://www.mdpi.com/article/10.3390/ijerph20032083/s1, Table S1: CONSORT Checklist of information to include when reporting randomized crossover trials.
