# Supplementary material for: Time of Day and Chronotype-Dependent Synchrony Effects Exercise-Induced Reduction in Migraine Load: A Pilot Cross-Over Randomized Trial
Source: Int J Environ Res Public Health. 2023 Jan 23;20(3):2083. doi: 10.3390/ijerph20032083 (PMC9915413; doi:10.3390/ijerph20032083)
Supplement: Supplementary file 1 [file ijerph-20-02083-s001.zip › ijerph-2170835-supplementary.pdf]

Supplementary Table S1. CONSORT Checklist of information to include when reporting randomized crossover trials.

Section/topic	Item No	Description	Page No
Title	1a	Identification as a randomized crossover trial in the title	1
Abstract	1b	Specify a crossover design and report all information outlined in table 2	1
Introduction:			
Background	2a	Scientific background and explanation of rationale	1-2
Objectives	2b	Specific objectives or hypotheses	2
Methods:			
Trial design	3a	Rationale for a crossover design. Description of the design features including allocation ratio, especially the number and duration of periods, duration of washout period, and consideration of carryover effect	2-3
Change from protocol	3b	Important changes to methods after trial commencement (such as eligibility criteria), with reasons	4
Participants	4a	Eligibility criteria for participants	2
Settings and location	4b	Settings and locations where the data were collected	3
Interventions	5	The interventions with sufficient details to allow replication, including how and when they were actually administered	3
Outcomes	6a	Completely defined pre-specified primary and secondary outcomes measures, including how and when they were assessed	3
Changes to outcomes	6b	Any changes to trial outcomes after the trial commenced, with reasons	4
Sample Size	7a	How sample size was determined, according for within participant variability	4
Interim analyses and stopping guidelines	7b	When applicable, explanation of any interim analyses and stopping guidelines	4
Randomization:			
Sequence generation	8a	Method used to generate the random allocation sequence	2
Sequence generation	8b	Type of randomization; details of any restriction (such as blocking and block size)	2-3,10
Allocation concealment mechanism	9	Mechanism used to implement the random allocation sequences (such as sequentially numbered containers), describing any steps taken to conceal the sequence until interventions were assigned	2-3,10
Implementation	10	Who generated the random allocation sequence, who enrolled participants, and who assigned participants to the sequence of interventions	2
Blinding	11a	If done, who was blinded after assignment to interventions (for example, participants, care providers, those assessing outcomes) and how	3
Similarity of interventions	11b	If relevant, description of the similarity of interventions	3, 5-6
Statistical methods	12a	Statistical methods used to compare groups for primary and secondary outcomes which are appropriate for crossover design (that is, based on within participant comparisons)	4
Additional analyses	12b	Methods for additional analyses, such as subgroup analyses and adjusted analyses	4
Results:			
Participant flow	13a	The numbers of participants who were randomly assigned, received intended treatment, and were analyzed for the primary outcome, separately for each sequence and period	4
Losses and exclusions	13b	No of participants excluded at each stage, with reasons, separately for each sequence and period	4-5
Recruitment	14a	Dates defining the periods of recruitment and follow-up	3
Trial end	14b	Why the trial ended or was stopped	4
Baseline data	15	A table showing baseline demographic and clinical characteristics by sequence and period	5

Numbers analyzed	16	Number of participants (denominator) included in each analysis and whether the analysis was by original assigned groups	4
Outcomes and estimation	17a	For each primary and secondary outcome, results including estimated effect size and its precision (such as 95% confidence interval) should be based on within participant comparisons. In addition, results for each intervention in each period are recommended	5-8
Binary outcomes	17b	For binary outcomes, presentation of both absolute and relative effect sizes is recommended	8
Ancillary analyses	18	Results of any other analyses performed, including subgroup analyses and adjusted analyses, distinguishing pre-specified from exploratory	5-8
Harms	19	Describe all important harms or unintended effects in a way that accounts for the design (for specific guidance, see CONSORT for harms	5
Discussion:			
Limitations	20	Trial limitations, addressing sources of potential bias, imprecision, and if relevant, multiplicity of analyses. Consider potential carry over effects.	10
Generalizability	21	Generalizability of the trial findings	10
Interpretation	22	Interpretation consistent with results, balancing benefits and harms, and considering other relevant evidence	9-10
Other information:			
Registration	23	Registration number and name of trial registry	11
Protocol	24	Where the full trial protocol can be accessed, if available	11
Funding	25	Sources of funding and other support (such as supply of drugs), role of funders	11
